# Combined plasma gas-phase synthesis and colloidal processing of InP/ZnS core/shell nanocrystals

**DOI:** 10.1186/1556-276X-6-68

**Published:** 2011-01-12

**Authors:** Ryan Gresback, Ryan Hue, Wayne L Gladfelter, Uwe R Kortshagen

**Affiliations:** 1 Department of Mechanical Engineering, University of Minnesota, Minneapolis, MN, USA; 2 Department of Chemistry, University of Minnesota, Minneapolis, MN, USA

## Abstract

Indium phosphide nanocrystals (InP NCs) with diameters ranging from 2 to 5 nm were synthesized with a scalable, flow-through, nonthermal plasma process at a rate ranging from 10 to 40 mg/h. The NC size is controlled through the plasma operating parameters, with the residence time of the gas in the plasma region strongly influencing the NC size. The NC size distribution is narrow with the standard deviation being less than 20% of the mean NC size. Zinc sulfide (ZnS) shells were grown around the plasma-synthesized InP NCs in a liquid phase reaction. Photoluminescence with quantum yields as high as 15% were observed for the InP/ZnS core-shell NCs.

## Introduction

Over the last two decades, semiconductor nanocrystals (NCs) have attracted significant attention because of their various unique properties. Semiconductor NCs provide size-tunable optical and electrical properties, based on quantum confinement of charge carriers within them, high surface-to-volume ratios, and other attributes that have led to the development of a new generation of materials and devices [1,2]. A significant amount of study has focused on compound semiconductor NCs of the II-VI and IV-VI systems because of the relative ease of synthesizing high quality materials using colloidal techniques [3,4]. While synthesis and surface functionalization methods for these NC materials are well established and NCs have shown impressive optical and electronic properties, there exists considerable interest to produce systems consisting of NCs of other materials whose constituent elements pose less of an environmental concern, which are more radiation resistant, and less prone to photo-oxidation.

Compound semiconductor NCs of the III-V systems may offer some of these desirable attributes. While III-V semiconductors are known to be "radiation-hard," they also are direct bandgap semiconductors, which is a big advantage for optical applications compared to, for instance, group IV NC materials [5]. Hence, there has been considerable interest in the synthesis of high quality III-V compound NCs, such as indium phosphide (InP). While synthesizing methods similar to those of II-VI semiconductor NCs can be applied to InP NCs, these have turned out to be very difficult and time consuming, often requiring days to produce NCs of high quality [6,7]. More recently, synthesis of high quality InP NCs has been achieved with non-coordinating solvents [8] and weak-coordinating solvents [9]. However, as these synthesizing methods require organic surfactants to stabilize NCs in solution during synthesis, they provide challenges for applications of the synthesized NCs in devices because of the often electrically insulating nature of the ligands.

Synthesis of NCs using nonthermal plasmas has become a viable method even for materials, which need to be produced in crystalline form requiring high temperatures, such as Si [10,11] and Ge [12]. An added advantage of plasma synthesis as compared to liquid phase routes is that the resulting material is free of ligands or surfactants, and that the ligands can be added later depending on the targeted application. This makes integration of plasma-produced NCs into devices easier, as it eliminates intermediate steps of ligand exchanges often required for II-VI and IV-VI NCs. In this article, we propose a method to synthesize InP NCs with a nonthermal plasma. In addition, we show that solution chemistry routes can be used for *post-synthesis *functionalization of the NCs with ligands and for growing an inorganic zinc sulfide (ZnS) shell around the plasma-synthesized InP NCs.

## Experimental

**Figure 1:**
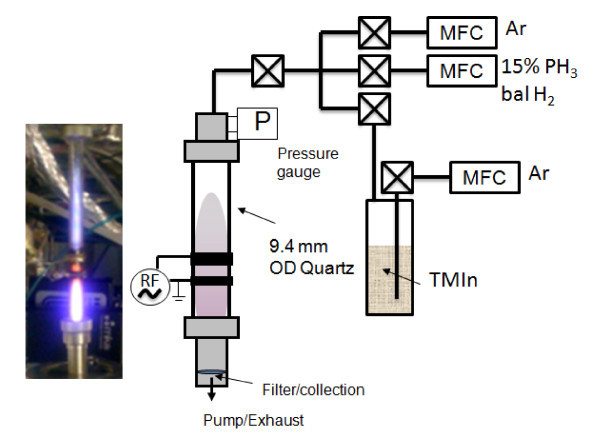
**Nonthermal plasma synthesis of InP NCs**. (left) Photograph and (right) schematic representation.

InP NCs were synthesized using a nonthermal radio-frequency (13.56 MHz) plasma operated at 2.5 Torr with nominal power ranging from 50 to 80 W applied through a matching network. A system schematic is shown in Figure 1. The reactor consists of two ring electrodes around a 9.4-mm-outer diameter quartz tube with a 6.25-mm inner diameter. Similar reactor designs were reported for the synthesis of Si [10] and Ge NCs [12]. The ground and powered electrodes were, respectively, located 3 and 4 cm from the grounded metal fitting. A precursor gas mixture of 15% phosphine (PH_3_) in hydrogen (H_2_) and trimethylindium (TMIn, In(CH_3_)_3_) vapor was introduced into the reactor. TMIn sublimes at room temperature allowed its vapor to be entrained in an argon carrier gas stream [13]. Additional argon is used to sustain the plasma and dilute the precursors. This study used a ratio of 90 Ar:17 H_2_:3 PH_3_:1 TMIn, with total mass flow rates being varied between 34 and 172 sccm, which resulted in residence times of the gas within the plasma zone to be between 2 and 10 ms.

The precursors were dissociated in the plasma by the hot plasma electrons, leading to nucleation and growth of InP NCs. The NCs were transported out of the plasma region by gas drag and deposited on a stainless steel mesh as a dry powder. The powder was extremely sensitive to air exposure; therefore, all the processes were performed under the exclusion of oxygen and moisture. After the synthesis, the NC material was transferred under nitrogen to a glove box or Schlenk line for handling and further reactions. The NCs produced in the plasma were found to react readily with ligands and coordinating solvents, such as amines, phosphine oxides, and fatty acids in the presence of non-coordinating solvents at temperatures less than 200°C in less than 1 h.

Based on the article by Xie et al. [14], the ZnS shells were grown as follows: a known mass of dry InP NCs was transferred into a solution of octadecene (ODE) and myristic acid under nitrogen. Then, the solution was sonicated for 5 min and heated to 200°C for 30 min under a flow of nitrogen. Each monolayer of ZnS was grown by using preheated and deoxygenated solutions of 0.05 M zinc stearate in ODE and sulfur in ODE. The zinc and sulfur precursors were injected separately at 15-min intervals with stoichiometric ratios (based on a spherical volume thickness calculation) and heated to 220°C. The dispersion in ODE was then cooled and repeatedly washed with methanol, precipitated by the addition of acetone, and separated by centrifugation. The InP/ZnS NCs were then re-dispersed in toluene.

Transmission electron microscopy (TEM) was performed by dropping a small amount of the InP colloid onto a thin carbon-coated TEM grid. A FEI Tecnai T12 (FEI Company, Hillsboro, OR, USA) operated at 120 kV was used. X-ray diffraction (XRD) was performed on a Bruker-AXS Microdiffractometer (Bruker Scientific Instruments, Billerica, MA, USA) with a 2.2-kW sealed Cu X-ray source. Raman spectroscopy was performed using a Witec Alpha300R (WITec GmbH, Ulm, Germany) confocal Raman microscope. Samples for XRD and Raman spectroscopy were prepared by casting drops of concentrated solutions onto glass substrates. Photoluminescence (PL) measurements were preformed on a Photon Technology International QuantaMaster 40 UV-Vis (Photon Technology International, Inc. Birmingham, NJ, USA) spectrofluorometer. UV-Vis absorption measurements were performed using a HR2000 spectrometer (Ocean Optics, Inc. Dunedin, FL, USA) with a combination of deuterium and tungsten halogen lamps (DH-2000-BALL).

## Results and discussion

**Figure 2:**
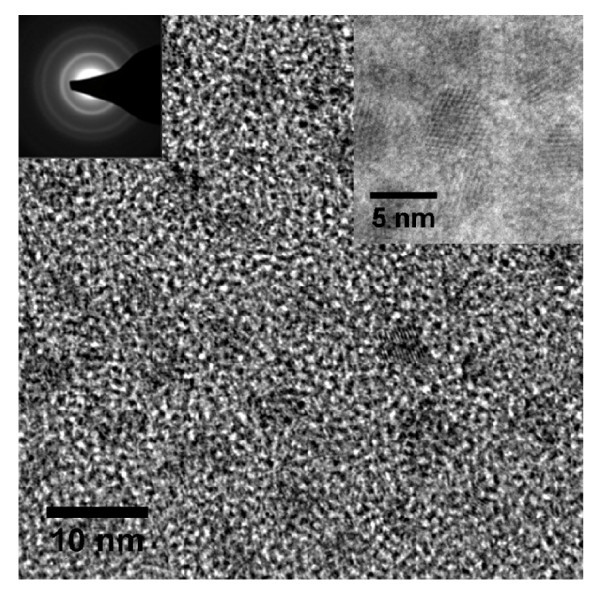
**TEM image of InP NCs synthesized with a 10-ms residence time. The average size is 4.3 nm**. Insets: SAD showing (111), (220), and (311) planes and HR image.

Bare InP NCs were found to oxidize rapidly making characterization of dry powder difficult. Hence, organic ligands are used to inhibit oxidation, afford dispersibility, and to allow for subsequent material characterization. Figure 2 shows a TEM image of InP NCs synthesized with a 10 ms residence time and passivated with myristic acid. The (111) lattice fringes are clearly visible in several NCs. The inset in Figure 2 shows a selected area diffraction (SAD) pattern with the rings of the (111), (220), and (311) InP planes being clearly visible. The average NC size determined by counting more than 50 particles was 4.3 nm with a standard deviation of 0.8 nm. The size distribution is broad compared with liquid-phase synthesis techniques, where a standard deviation of less than 10% of the average size is common [14]; however, with a standard deviation of approximately 20% of the mean size, the size distribution is rather narrow for gas-phase synthesized NCs [15].

**Figure 3:**
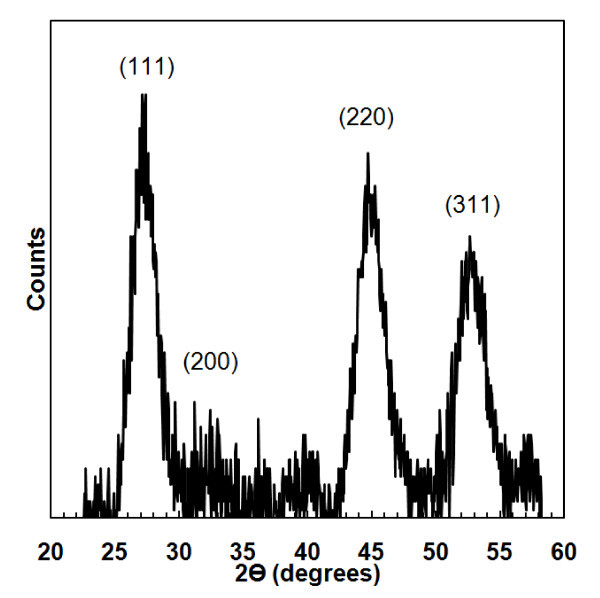
**XRD spectrum of InP NCs synthesized with a 6-ms residence time. The size given by the Debye-Scherrer equation is 3.6 nm**.

**Figure 4:**
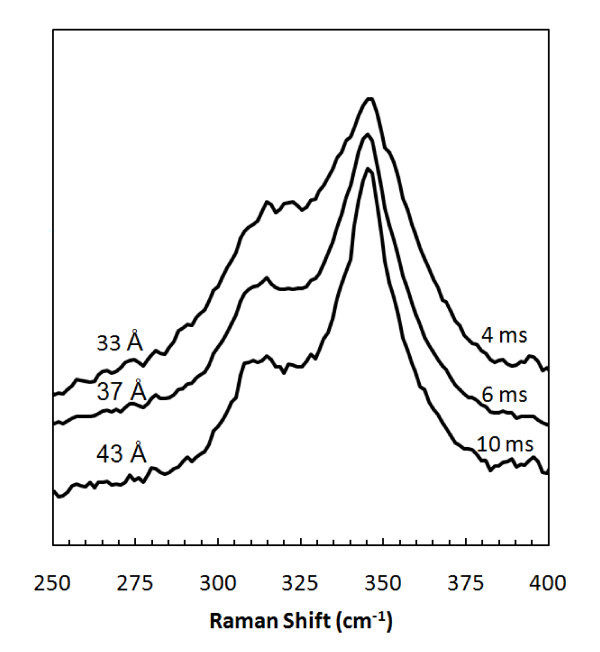
**Raman spectra of InP NCs passivated with myristic acid and synthesized using various plasma residence times; NC diameters from TEM**.

Raman spectroscopy and XRD were used to investigate further the NC microstructure and size. Figure 3 shows an XRD spectrum of NCs synthesized with a residence time of 6 ms. The (111), (220), and (311) peaks are clearly visible with a small signal from the (200) peak. This confirms the presence of InP with a zinc-blend structure. Debye-Scherrer analysis of XRD peak broadening gives a NC size of 3.4 nm, while TEM results found a slightly larger mean size of 3.8 nm. The Raman spectra in Figure 4 shows InP NCs synthesized with residence times of 4, 6, and 10 ms and passivated with myristic acid. The peak of the longitudinal optical mode decreases while the transverse optical mode increases, with decreasing residence time and NC size. This was also observed by Guzelian et al. [16] and was thought to be because of a loss of symmetry as NC size decreases.

**Figure 5:**
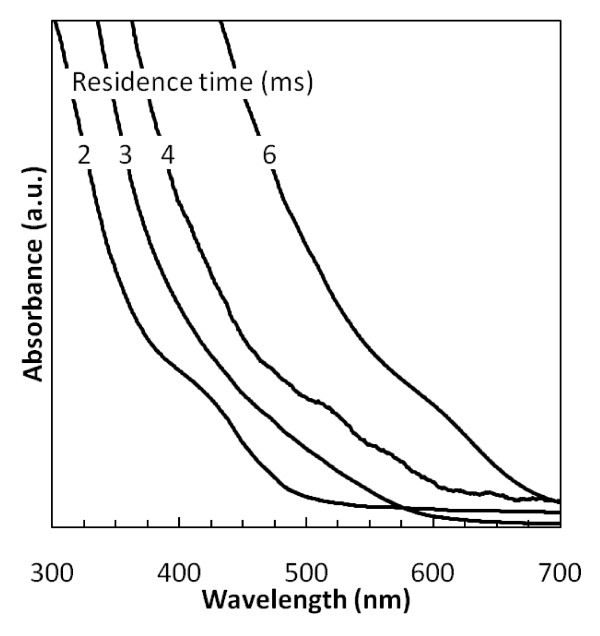
**UV-Vis absorption spectra of InP NCs passivated with myristic acid synthesized using various residence times in the plasma**.

UV-Vis absorption was performed on colloidal InP NCs with myristic acid as the ligand after being heated to 190°C for 1 h. Figure 5 shows that the onset of the absorption changes as a function of the residence time of the NCs in the plasma. Shorter residence times lead to a blue shift in the onset of the absorption, corresponding to smaller NCs. The first exciton peak is not as clearly defined as in solution-synthesized InP NCs, likely because of the broad size distribution. The onset of the absorption correlates well with sizes observed in TEM.

**Figure 6:**
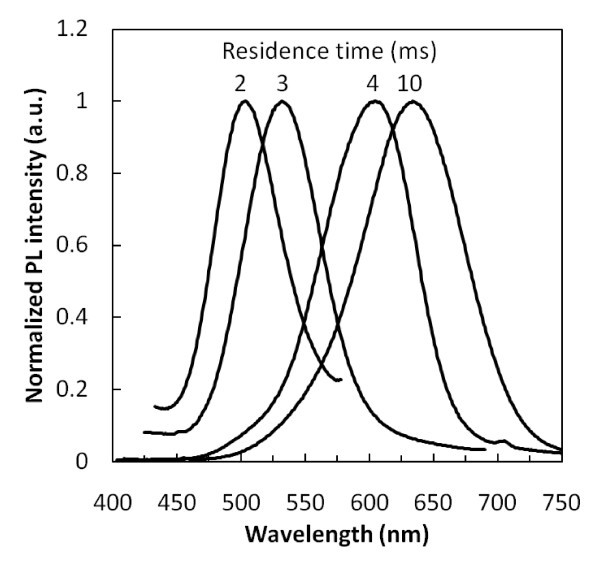
**Representative PL spectra of InP/ZnS core/shell NCs synthesized with various plasma residence times**.

**Figure 7:**
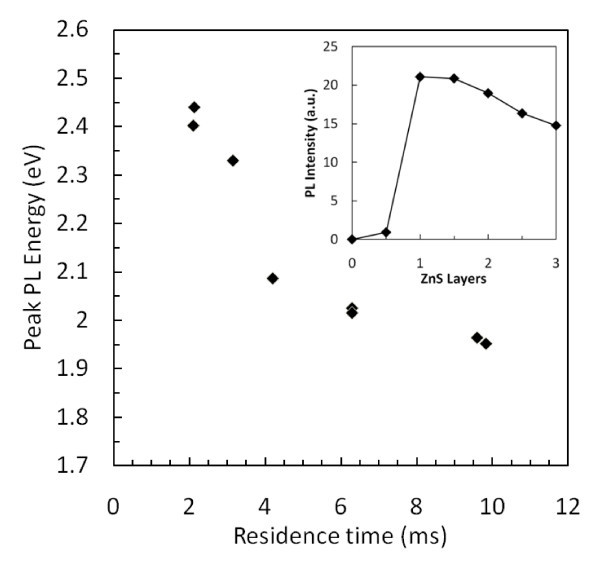
**Effect of plasma residence time on peak PL emission from InP/ZnS core/shell structures**. Inset: Layer-by-layer effect of ZnS growth on PL intensity where whole layers represent a full cycle of Zn and S injections.

Both the as-produced NCs and the NCs solely functionalized with organic ligands show little or no PL. The NCs that were allowed to oxidize exhibit a small increase in PL; however, the quantum yield (QY) of these samples was still less than 1%. Low QY in InP NCs is often attributed to phosphorus-dangling bonds at the surface which lead to non-radiative recombination of photo-generated carriers [17]. Several methods were proposed to eliminate non-radiative recombination sites, including fluorine etches [17] and capping of NCs with a wideband gap semiconductor such as ZnS [14]. In this study, we used the method of growing a ZnS layer. Figure 6 shows the representative PL spectra of several samples of InP/ZnS core-shell NCs. Peak emission from the InP/ZnS NCs varies from 508 to 635 nm with the full-width half-maximum of the PL varying between 70 and 100 nm. The peak emission, like the absorption onset, is strongly dependent on the residence times of the NCs in the plasma. Figure 7 shows the relationship between the residence time and peak emission. Unfortunately, owing to experimental setup limitations, a wider range of residence time could not be explored. However, there is no indication that a continuous range of emission from bulk emission at 1.37 eV to quantum-confined NC emission at 2.5 eV could not be achieved. As is shown in the inset of Figure 7, it is remarkable to see that a single monolayer of ZnS leads to a significant increase of the PL QY. The addition of more ZnS monolayers does not lead to a further increase in PL, likely because all non-radiative states were passivated by the first monolayer.

Quantum yields between 10 and 15% were measured for InP/ZnS structures. This is lower than the best results reported by Xie et al. [14] for solution-grown InP capped by ZnS using zinc stearate and elemental sulfur, and the cause for this difference is unknown. In future studies, the authors will explore the use of more reactive precursors, such as diethylzinc and *bis*(trimethylsilyl)sulfide, which have also been found effective for coating solution-grown InP NCs resulting in higher emission QYs [18].

## Conclusion

In summary, InP NCs with controllable size were successfully synthesized from the flow-through nonthermal plasma. Size control was achieved through adjusting the residence time of NCs in the plasma. This synthesis process yields NCs which are bare and free-standing, which may simplify device integration. In addition, we find that these NCs can easily form a colloid with a variety of ligands, which then enables application of well-established colloidal-processing techniques. After capping the InP NCs with a ZnS shell, PL QYs between 10 and 15% were observed. This study has demonstrated that it is possible to synthesize compound semiconductor NCs with plasma, opening up the possibility of a wider range of the plasma-synthesized NC materials.

## Competing interests

The authors declare that they have no competing interests.

## Authors' contributions

RG synthesized the InP quantum dots, contributed to the ZnS shell functionalization, and drafted the manuscript. RH contributed to the ZnS shell functionalization. UK and WLG were involved in the scientific guidance of the research and in revising the manuscript.
